# The Toxicokinetic Profile of Dex40-GTMAC3—a Novel Polysaccharide Candidate for Reversal of Unfractionated Heparin

**DOI:** 10.3389/fphar.2016.00060

**Published:** 2016-03-17

**Authors:** Emilia Sokolowska, Bartlomiej Kalaska, Kamil Kaminski, Alicja Lewandowska, Agnieszka Blazejczyk, Joanna Wietrzyk, Irena Kasacka, Krzysztof Szczubialka, Dariusz Pawlak, Maria Nowakowska, Andrzej Mogielnicki

**Affiliations:** ^1^Department of Pharmacodynamics, Medical University of BialystokBialystok, Poland; ^2^Faculty of Chemistry, Jagiellonian UniversityKrakow, Poland; ^3^Department of Histology and Cytophysiology, Medical University of BialystokBialystok, Poland; ^4^Department of Experimental Oncology, Ludwik Hirszfeld Institute of Immunology and Experimental Therapy, Polish Academy of SciencesWroclaw, Poland

**Keywords:** dextran, heparin, protamine sulfate, safety, toxicity, toxicokinetics

## Abstract

Though protamine sulfate is the only approved antidote of unfractionated heparin (UFH), yet may produce life threatening side effects such as systemic hypotension, catastrophic pulmonary vasoconstriction or allergic reactions. We have described 40 kDa dextrans (Dex40) substituted with glycidyltrimethylammonium chloride (GTMAC) as effective, immunogenically and hemodynamically neutral inhibitors of UFH. The aim of the present study was to evaluate in mice and rats toxicokinetic profile of the most promising polymer—Dex40-GTMAC3. Polymer was rapidly eliminated with a half-time of 12.5 ± 3.0 min in Wistar rats, and was mainly distributed to the kidneys and liver in mice. The safety studies included the measurement of blood count and blood biochemistry, erythrocyte osmotic fragility and the evaluation of the histological alterations in kidneys, liver and lungs of mice and rats in acute and chronic experiments. We found that Dex40-GTMAC3 is not only effective but also very well tolerated. Additionally, we found that protamine may cause overt hemolysis with appearance of permanent changes in the liver and kidneys. In summary, fast renal clearance behavior and generally low tissue accumulation of Dex40-GTMAC3 is likely to contribute to its superior to protamine biocompatibility. Intravenous administration of therapeutic doses to living animals does not result in the immunogenic, hemodynamic, blood, and organ toxicity. Dex40-GTMAC3 seems to be a promising effective and safe candidate for further clinical development as new UFH reversal agent.

## Introduction

Unfractionated heparin (UFH) is mainly utilized in hospitals during the treatment and prevention of thrombosis, but is associated with an increased risk of bleeding and other adverse effects. Although, UFH is being replaced by low molecular weight heparins (LMWHs) or fondaparinux because of the safety reasons, according to the latest reports the Europe heparin market is estimated to be around USD 2 billion and may reach USD 3 billion in 2022; UFH share is still at around 10% (Transparency Market Research, 2015). In contrast to the new parenteral anticoagulants, UFH is inexpensive and it can be fully neutralized by protamine sulfate in case of bleeding. Importantly, UFH, discovered a century ago is one of the oldest drugs, with very well defined advantages, side effects, and therapeutic applications (Wardrop and Keeling, 2008). Indeed, the use of UFH and protamine is essential during many cardiovascular procedures, such as coronary artery bypass, heart transplant, cardiac valve repair or repair of congenital heart problems; their number increased by 28% from 5, 939, 000 in 2000 to 7, 588, 000 in 2010 in USA (Mozzaffarian et al., 2015). Also, surgical procedures like aortic aneurysm repair, arteriovenous fistula grafts for hemodialysis, carotid endarterectomy, complex vascular reconstruction or femoral popliteal bypass often require co-administration of UFH and protamine (Mahan, 2014).

Protamine sulfate, the only approved antidote for heparin-based therapies, is a purified mixture of cationic proteins obtained from the sperm of wild salmon-like species fished over a specific area off the coast of Honshu Island. This cationic peptide binds to heparins, which are negatively charged, to form a complex that lacks anticoagulant activity. Despite poor therapeutic index, protamine has been used with caution since 1939, because there is no alternative. Many methods, such as administration of small or divided doses were tried to prevent the adverse effects of protamine, which include systemic hypotension, pulmonary vasoconstriction and allergy reactions, sporadically leading to cardiovascular collapse and death (Hirsh et al., 2008). The incidence of mild reactions to protamine is as high as 16% and that of severe reactions is between 0.2 and 3.0% (Lowenstein et al., 1983; Holland et al., 1984; Katz et al., 1987; Cook et al., 1992). Additionally, unseen pathological effects of protamine may contribute to the postsurgical morbidity and mortality. Therefore, many studies were undertaken in the past to develop safer replacer of protamine (Bromfield et al., 2013). The most advanced agents are in the animal (universal heparin reversal agents, UHRAs) (Shenoi et al., 2014) or human (idarucizumab) (Pollack et al., 2015) phases of drug development; andexanet alfa (Lu et al., 2013) and aripazine (PER977) (Ansell et al., 2014) already finished first clinical trials and received breakthrough designation from the FDA. In clinical trials patients' safety is paramount, therefore potential toxicity of new candidates for drugs is the most important issue. A number of compounds that have been primarily introduced as very effective antidotes of heparins such as delparantag (PMX-60056) (Mahan, 2014), REG1 Anticoagulation System (Burke, 2015), PM102 (Cushing et al., 2010), virus-like nanoparticles (VLP) (Gale et al., 2011) or HepArrestTM (Shenoy et al., 1999), later in the preclinical and even in the clinical trials revealed unacceptable side effects and failed.

We have recently presented series of different synthetic water-soluble molecules that directly complex UFH (Kaminski et al., 2008, 2010, 2011, 2014; Kalaska et al., 2012, 2015). Dextran with 40 kDa of mean molecular weight substituted with GTMAC at a ratio of 0.65 GTMAC groups per a glucose unit (Dex40-GTMAC3), originated from an FDA-approved, generally nontoxic, biodegradable and inexpensive polysaccharide, did not elicit detectable immune response at the doses that allowed complete neutralization of UFH at *in vitro* and *in vivo* conditions (Kalaska et al., 2015). The aim of this study was to provide a toxicokinetic profile of intravenously injected Dex40-GTMAC3. We evaluated the safety profile in mice and rats using different approaches to explore the possible application for restoring normal blood clotting in heparinized patients.

## Materials and methods

### Animals and housing

Animals were purchased and housed in the Centre of Experimental Medicine of Medical University of Bialystok in specific pathogen free conditions according to Good Laboratory Practice rules. Thirty-two male Wistar rats, 34 male NMRI-Foxn1nu/Foxn1nu mice and 10 BALB/c male mice were used in all experiments. Animals were housed with a 12 h light/dark cycle in temperature (22 ± 2°C) and humidity (55 ± 5%) controlled room, grouped in cages as appropriate, and allowed to have *ad libitum* access to sterilized tap water and a standard chow (Ssniff R-Z V1324). The animals' health status was monitored throughout the experiments by a health surveillance programme according to Federation of European Laboratory Animal Science Associations (FELASA) guidelines. The rats and mice were free of all viral, bacterial, and parasitic pathogens listed in the FELASA recommendations. All the procedures involving animals and their care were approved by Local Ethical Committee on Animal Testing at the Medical University of Bialystok (Permit Numbers 28/2012 and 15/2013) and by First Local Ethical Committee on Animal Testing at the Polish Academy of Science in Wroclaw (Permit Number 26/2014) and conducted in accordance with ARRIVE guidelines (McGrath et al., 2010), directive 2010/63/EU of the European Parliament and of the Council on the protection of animals used for scientific purposes and the national laws. Procedures were conducted in the light phase of cycle in the surgical room of our laboratory. All animals were euthanized by pentobarbital injection at the end of experiments.

### Materials

Heparin sodium salt from bovine intestinal mucosa (UFH), protamine (protamine sulfate salt from salmon, grade X), dextran (MW = 40 kDa from Leuconostoc spp.), sodium chloride (analytical grade), rhodamine B isothiocyanate (RBITC, HPLC grade), fluorescein isothiocyanate (FITC, HPLC grade), glycidyltrimethylammonium chloride (GTMAC, technical grade), sodium hydroxide (analytical grade), acetone (analytical grade), pyridine (analytical grade), dimethyl sulfoxide (analytical grade), dipotassium ethylenediaminetetraacetic acid (K_2_EDTA, analytical grade) were purchased from Sigma-Aldrich (Germany). Isoflurane was purchased from Baxter (Germany). Pentobarbital, ketamine, xylazine were purchased from Biovet Pulawy (Poland). Trisodium citrate was purchased from Avantor Performance Materials, Gliwice, Poland). Ethanol 96% was used in the study. Dex40-GTMAC3 was synthesized as described previously (Kaminski et al., 2011; Kalaska et al., 2015). Dex40-GTMAC3 was fluorescently labeled with RBITC and FITC as follows. Two hundred and fifty mg of polymer was dissolved in 20 ml of warm DMSO and 3 drops of pyridine and 15 mg of a respective isothiocyanate was added. The mixture was heated to 95°C for 2 h under continuous stirring. After this time DMSO was removed by dialysis against water for 6 h, then the product was precipitated with acetone. The degree of substitution with the fluorescent probe was ~1% per glucose unit.

### The measurement of fluorescein-labeled Dex40-GTMAC3 concentration in rats

**Figure 1:**
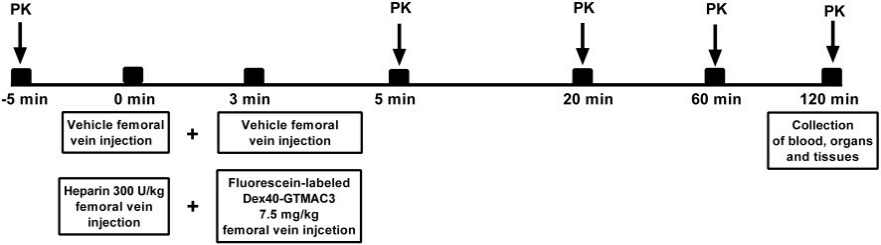
**Schematic representation of the study protocol**. PK = the measurement of plasma protamine/fluorescein-labeled Dex40-GTMAC3 for pharmacokinetic characterisation.

Three male Wistar rats weighting 213 ± 3 g were anesthetized by an intraperitoneal injection of pentobarbital (45 mg/kg b.w.) and placed in a supine position on a heated operation table. Heparin (300 U/kg) or vehicle (PBS) pre-treatment was followed after 3 min with fluorescein-labeled Dex40-GTMAC3 administration via femoral vein in dose of 7.5 mg/kg (Figure 1). Approximately 500 μl of blood was collected via common carotid artery 5 min prior and at 5, 20, 60, and 120 min following heparin or vehicle injection to measure plasma Dex40-GTMAC3 concentration for pharmacokinetic assessment. Animals were sacrificed at 120 min by the injection of pentobarbital (45 mg/kg b.w.), blood samples were taken from the heart and organs (liver, small and large intestine, kidneys, lungs, heart, abdominal aorta, left and right nucleus, bladder, brain) were collected, weighed, and frozen at −80°C. Then, organs were homogenized using an ultrasonic homogenizer (UP50H Dr. Hielscher GmbH) 50W 33Hz in PBS buffer at a pH of 7.4 at a ratio of 1 g tissue and 2 ml buffer. Homogenates were centrifuged for 5 min at 6000 rpm. The supernatant was transferred to a cuvette and diluted 3 times. The measurement was performed at a wavelength of 485 nm excitation. T (terminal elimination half-life) was calculated by PK Solver Excel add-in program using Non-Compartmental Analysis of Plasma Data after Intravenous Bolus Input Model.

### The body distribution of rhodamine-labeled Dex40-GTMAC3 in mice

The body distribution of the cationic dextran was measured in 30 NMRI-Foxn1nu/Foxn1nu mice anesthetized with a mixture of isoflurane and oxygen at a concentration of 3% (maintained then at a concentration of 1.5–2.5%); 5 mice served as a control group. Rhodamine-labeled Dex40-GTMAC3 was administered in dose of 22.5 mg/kg b.w. 5 min after UFH (300 U/kg) to 25 mice and body distribution was measured in 5, 15, 30, 60, and 120 min (5 different animals for each time point) using *In-vivo* MS FX PRO system (Carestream Health INC., USA). The brain, lungs, kidneys, heart, liver and spleen were isolated from mice and their fluorescence was measured *ex vivo*. The signal intensity in control group (0.9, 0.9, 1.0, 1.2, and 1.0 I[AU] 10^7^ in livers, 0.2, 0.3, 0.2, 0.2, and 0.2 I[AU]•10^7^ in kidneys, 2.5, 2.8, 2.4, 2.0, and 1.6 I[AU]•10^6^ in lungs, 1.1, 1.5, 1.3, 1.1, and 1.3 I[AU]•10^6^ in spleens, 0.4, 0.7, 0.7, 0.9, and 0.5 I[AU]•10^6^ in hearts, 1.4, 2.8, 2.4, 2.5, and 2.5 I[AU]•10^6^ in brains, for 5, 15, 30, 60, and 120 min, respectively) was subtracted from the presented results.

### Erythrocyte osmotic resistance assay

The osmotic resistance was measured according to the method described by Hunter (1940) in our modification (Tankiewicz et al., 2005). Briefly, blood collected from heart of Wistar rats on K_2_-EDTA as anticoagulant was mixed gently for 30 min in room temperature with different concentrations of protamine (1, 10, 100 μg/ml) or Dex40-GTMAC3 (1, 10, 100 μg/ml). Then, 10 μl of blood was added to 2 ml saline solution in range of concentration from 10 to 150 mM. The suspension was allowed to stand at room temperature for 30 min and was centrifuged at 1000 g for 5 min at 4°C. The absorbance of received supernatants was determined in microplate reader (Dynex Tech., USA) at 540 nm. Mean osmotic resistance (MOR50) is the concentration of NaCl, at which 50% of red cells were lysed.

### Collection of organs in short-term observation in mice

Ten male BALC/c mice weighing 24.5 ± 0.6 g (no significant difference between groups) and aged 8–10 weeks were randomly divided into 4 groups (2–3 per experimental group), anesthetized with a mixture of ketamine (125 mg/kg b.w.) and xylazine (12.5 mg/kg b.w.). UFH was administered into the tail vein in dose of 300 U/kg b.w. alone or followed (3 min) by intravenous administration of Dex40-GTMAC3 (7.5 mg/kg b.w.) or protamine (3 mg/kg b.w.). Fragments of organs (liver, kidney and lungs) were collected 1 h after drug administration, fixed in Bouin's fluid and processed routinely for embedding in paraffin. Sections were cut at 4 μm in thickness, and stained by hematoxylin and eosin (H&E) for routine histological examination.

### Histological evaluation of liver and lungs in long-term observation

**Figure 2:**
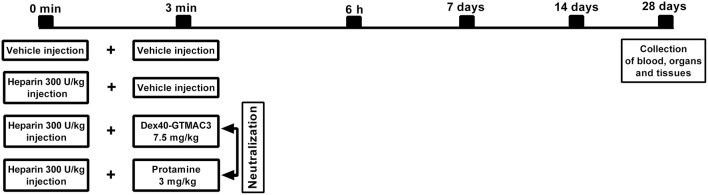
**Schematic representation of the study protocol**. Diagram shows the time course of events from vehicle or UFH administration following neutralization with Dex40-GTMAC3 or protamine injected in rat tail vein through blood sampling time points to the end of the experiment with the collection of organs.

Twenty-seven male Wistar rats weighing 220–300 g were anesthetized with a mixture of isoflurane and oxygen at a concentration of 2–4% (maintained then at a concentration of 1.5–2.5%). Animals were randomly divided into 4 groups (5–7 per experimental group). UFH was administered into the tail vein in dose of 300 U/kg b.w. alone or followed (3 min) by intravenous administration of Dex40-GTMAC3 (7.5 mg/kg b.w.) or protamine (3 mg/kg b.w.) (Figure 2). Vehicle (PBS) treated rats served as a control group. Then, after 6 h, 7, 14, 28 days, the animals were weighed, again anesthetized with a mixture of isoflurane and oxygen, and 0.3 ml of blood was collected from the tail artery on the standard anticoagulant for the evaluation of hematological parameters: white blood cells (WBC), red blood cells (RBC), hemoglobin (HGB), hematocrit (HCT), mean corpuscular volume (MCV), mean corpuscular hemoglobin (MCH), mean corpuscular hemoglobin concentration (MCHC), platelets (PLT) in a blood analyzer (ABC Vet, Horiba, Germany). Zero point seven ml of blood was centrifuged and biochemical parameters: aspartate aminotransferase (AST), alanine aminotransferase (ALT), creatinine (CREA), amylase (AMYL), alkaline phosphatase (ALP), creatine kinase (CPK) were measured in serum by automated clinical biochemical analyzer (Mindray BS 120, Germany). After 28 days rats were anesthetized with pentobarbital at the dose of 45 mg/kg b.w. Blood was taken from the right ventricle to the standard anticoagulant. A part of blood was used for morphological analyses while the remaining amount has been used to receive plasma and serum, which were frozen at a temperature of −80°C. The same fragment of the liver lobe and kidney were collected from each rat for histological examination. The lungs were collected in whole. Immediately after thoracotomy, Bouin's fluid was administered with syringe into the trachea to smoothen the pulmonary pleura of isolated lungs. After fixation fragment of the lung (the anterior part of the superior pulmonary lobe) was collected.

### Histological evaluation of liver, kidneys, and lungs

All tissues were routinely placed in paraffin blocks and then sectioned by a Leica 2025 rotating microtome. Sections were cut into 4 μm in thickness, and later stained with hematoxylin and eosin (H&E) were evaluated under a light microscope.

### Statistical analysis

In the study n refers to the number of animals in each experimental group. For *in vivo* experiment, the experimental unit was an individual animal. The data were analyzed by GraphPad Prism 6 using the non-parametric Mann-Whitney test and presented as a median with lower and upper limits. Analysis of variance (ANOVA) was used to compare differences between means presented as mean ± SD, whenever data passed normality test. *P*-values less than 0.05 were considered significant. Half-time and C_0_ were estimated by non-compartmental methods (Beyerle et al., 2014). Fisher exact test with *post-hoc* Holm *p*-value adjustment was applied to compare proportions between groups pairwise (**Table 3**).

## Results

### The concentration of fluorescein-labeled Dex40-GTMAC3 in rats

**Figure 3:**
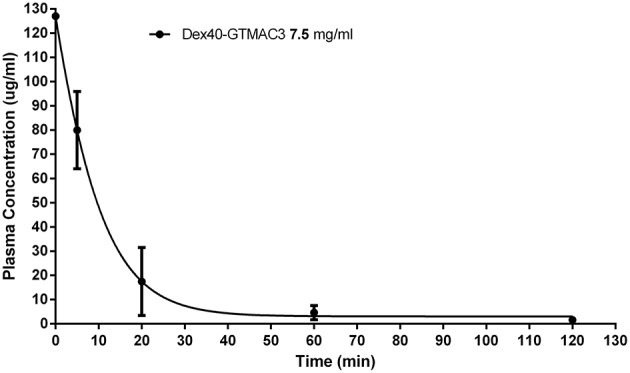
**Plasma concentration of Dex40-GTMAC3 (7.5 mg/kg) administered as an intravenous single injection 3 min following UFH (300 U/kg) administration**. Results are shown as mean ± SD.

The concentration of Dex40-GTMAC3 in plasma declined shortly after administration. After 60 min the amount of the circulating polymer was 5.8% of the amount measured in 5 min. T was 12.5 ± 3.03 min, whereas C_0_ was 126.7 μg/ml (determined automatically by extrapolation of the curve) (Figure 3). We found a signal of fluorescein-labeled Dex40-GTMAC3 in liver (1st rat = 38.90, 2nd rat = 13.34 and 3rd rat = 40.12) and kidneys (1st rat = 32.57, 2nd rat = 15.34 and 3rd rat = 61.34). High autofluorescence of heart and stomach in comparison to vehicle treated rats prevented the precise detection of polymer in these tissues. In the pancreas, spleen, brain, and lung fluorescence signal of fluorescein-labeled protein was below the detection limit.

### Distribution of rhodamine-labeled Dex40-GTMAC3 in mice

**Figure 4:**
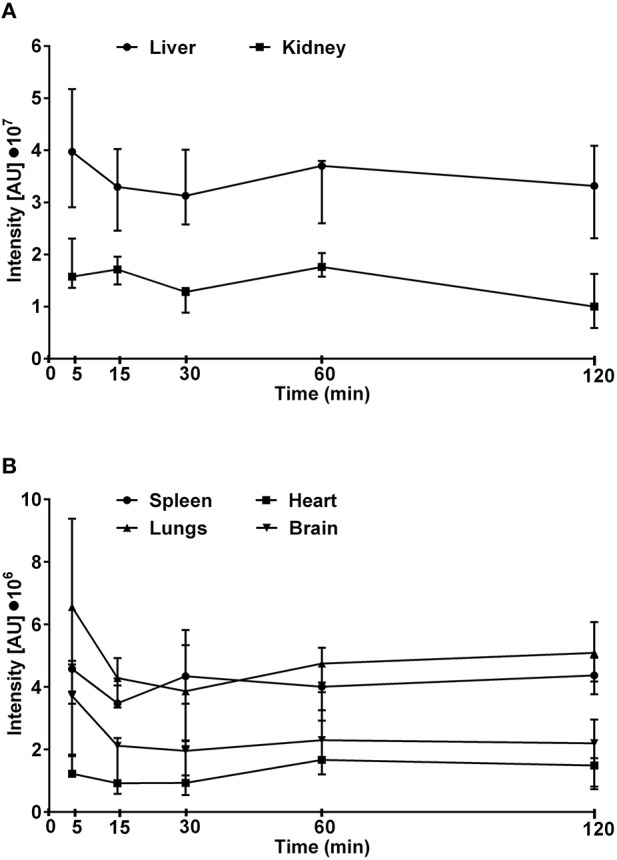
**The intensity of fluorescence measured in kidneys and liver (A) or lungs, heart, spleen, and brain (B) isolated from mice treated intravenously with rhodamine-labeled Dex40-GTMAC3 (22.5 mg/kg), 3 min following UFH (300 U/kg) administration**. The signal intensity in control group (0.9, 0.9, 1.0, 1.2, and 1.0 I[AU]•10^7^ in livers, 0.2, 0.3, 0.2, 0.2, and 0.2 I[AU]•10^7^ in kidneys, 2.5, 2.8, 2.4, 2.0, and 1.6 I[AU]•10^6^ in lungs, 1.1, 1.5, 1.3, 1.1, and 1.3 I[AU]•10^6^ in spleens, 0.4, 0.7, 0.7, 0.9, and 0.5 I[AU]•10^6^ in hearts, 1.4, 2.8, 2.4, 2.5, and 2.5 I[AU]•10^6^ in brains, for 5, 15, 30, 60, and 120 min, respectively) was subtracted from the presented results. Results are presented as median, upper/lower limits.

**Figure 5:**
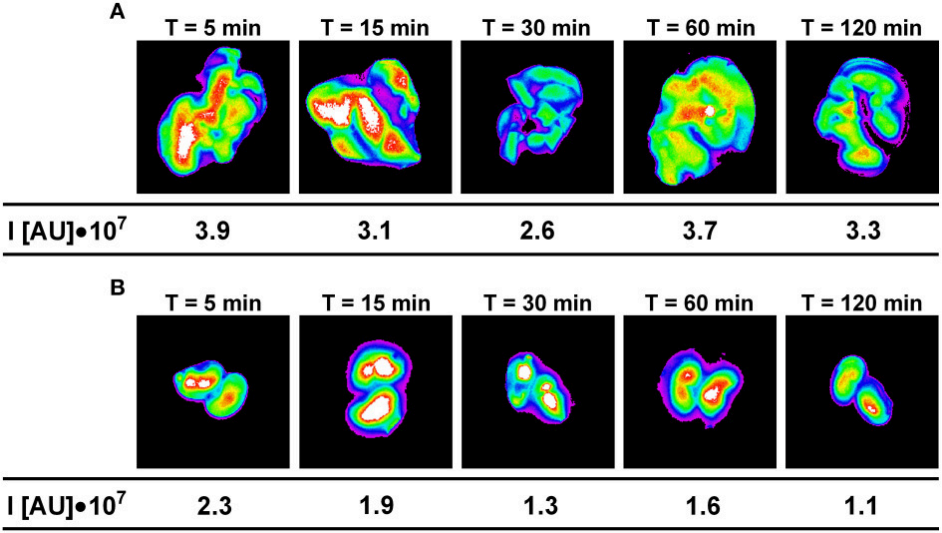
**The intensity of fluorescence in example livers (A) and kidneys (B) collected from mice treated intravenously with rhodamine-labeled Dex40-GTMAC3 (22.5 mg/kg), 3 min following UFH (300 U/kg) administration in the consecutive time points**. The signal intensity in control group (0.9, 0.9, 1.0, 1.2, and 1.0 I[AU]•10^7^ in livers and 0.2, 0.3, 0.2, 0.2, and 0.2 I[AU]•10^7^ in kidneys for 5, 15, 30, 60, and 120 min, respectively) was subtracted from the presented results.

The maximum fluorescence intensity was observed in the liver, kidneys and lungs 5 min after intravenous administration of Dex40-GTMAC3 (Figure 4A). The signal from liver and kidney of animals treated with rhodamine-labeled Dex40-GTMAC3 was higher than in vehicle-treated group (examples are presented in Figure 5). Then, the fluorescence in these organs decreased slightly, but remained at similar level for 2 h. Over the course of 120 min we found slight, but higher than recorded for vehicle treated animals signal of rhodamine-labeled Dex40-GTMAC3 localized in heart, brain, and spleen (Figure 4B).

### Influence of the Dex40-GTMAC3 on erythrocyte osmotic resistance

**Figure 6:**
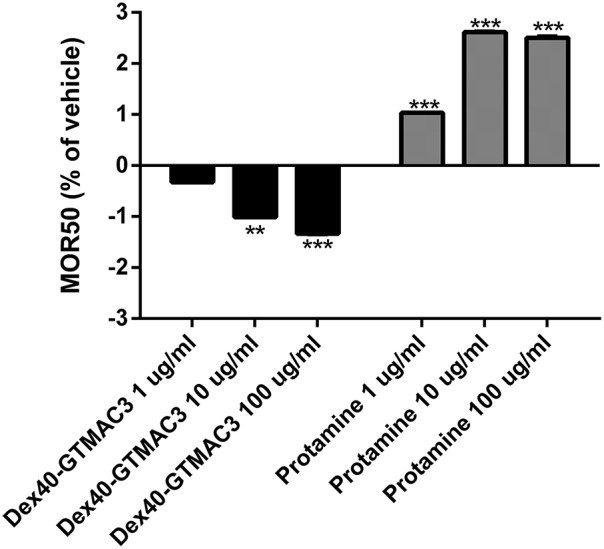
**Mean osmotic resistance (MOR50) exposed to Dex40-GTMAC3 and protamine**. Ten microliter of whole blood in 2 ml of increasing concentration of NaCl were incubated at room temperature in the absence (control) and in the presence of 1.0, 10, and 100 μg/ml tested compounds. Degree of hemolysis was calculated from the 540 nm absorbance of the supernatant after centrifugation of the erythrocyte suspensions. The results are expressed as mean percentage of total hemolysis in comparison to vehicle ± SD. ^**^p < 0.01, ^***^p < 0.001 vs. vehicle, Mann-Whitney test.

The initial hemolysis (>5%) occurred at 150 mmol/l saline solution in all samples. We observed a steadily increasing susceptibility to hypotonic lysis in blood samples incubated with increasing concentrations of test substances. Differences were most significant at NaCl concentrations of 80–90 mmol/l. MOR50 was 85.16 ± 3.82 in control group. Starting from concentration of 10 μg/ml Dex40-GTMAC3 significantly decreased MOR50, whereas protamine slightly, but significantly increased MOR50starting from concentration of 1 μg/ml (Figure 6).

### Influence of the Dex40-GTMAC3 on body weight

**Figure 7:**
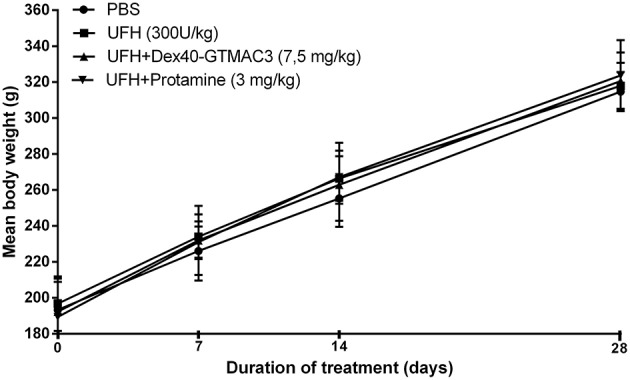
**Mean body weight from baseline to day 28**. Results are shown as mean ± SD.

No deaths were reported during 1 month observation. No drug-related clinical signs of toxicity, effects on food consumption or ocular changes were observed during the study. No significant differences were noted in mean baseline body weights between randomized treatment groups (Figure 7). The mean body weight for control rats increased from 193 ± 17.5 g at baseline to 314.7 ± 10.9 g at day 28 (mean body weight gain, 121.1 ± 16.6 g) and it was similar in rats receiving heparin (300 U/kg) followed by Dex40-GTMAC3 (7.5 mg/kg) (128.3 ± 9.8 g) or protamine (134.4 ± 25.5 g).

### Influence of the Dex40-GTMAC3 on blood parameters

WBC, RBC, HGB, HCT, MCV, MCHC, PLT were counted in whole blood following single intravenous administration of UFH (300 U/kg) alone or followed by Dex40-GTMAC3 (7.5 mg/kg) or protamine (3 mg/kg) after 7, 14, and 28 days. The injection of the tested compound did not change the blood parameters in all time points (Table 1). We found slight, but significant changes of some parameters between time points within groups as a result of differences in procedures, i.e., blood collection from rat tail vs. heart after chest opening. Furthermore, blood cell count in all experimental groups remained within the normal range (Giknis and Clifford, 2008).

**Table 1:** **Hematological analysis of whole blood samples from rats treated with UFH (300 U/kg) alone or followed by Dex40-GTMAC3 (7.5 mg/kg) or protamine (3 mg/kg)**.

**Parameters (unit)**	**Time (days)**	**Vehicle**	**UFH 300 U/kg**	**UFH 300 U/kg + Dex40-GTMAC3 7.5 mg/kg**	**UFH 300 U/kg + Protamine 3.0 mg/kg**
WBC (10^3^/mm^3^)	7	6.4 (6.3–13.0)	8.3 (6.7–12.5)	8.9 (5.2–13.2)	9.8 (6.5–15.3)
	14	10.5 (7.4–13.7)	12.6 (7.4–17.6)	10.3 (5.9–15.1)	9.2 (6.1–16.2)
	28	4.5^*^ (3.1–10.1)	6.0^*^^∧∧^ (3.7–7.9)	5.6^*^^∧^ (4.4–6.8)	5.2^**^^∧^ (3.9–6.5)
RBC (10^3^/mm^3^)	7	5.9 (5.2–6.6)	6.3 (5.8–6.5)	6.0 (4.9–6.9)	6.1 (5.7–6.4)
	14	6.7 (5.0–7.2)	6.7^*^ (6.0–7.3)	6.5 (5.2–6.3)	6.4 (6.1–6.7)
	28	7.1^*^ (7.0–7.4)	6.9^**^ (6.5–7.5)	7.0^**^ (6.6–7.8)	6.9^**^ (6.0–7.2)
HGB (g/dl)	7	13.6 (12.3–15.0)	14.0 (13.9–14.5)	14.0 (11.8–15.1)	14.1 (13.7–14.7)
	14	15.1^*^ (15.0–15.7)	14.7^*^ (14.3–15.6)	14.6 (11.7–16.3)	14.8 (14.0–15.4)
	28	14.7 (14.6–15.7)	14.6 (13.1–15.3)	14.8^*^ (14.5–15.9)	14.3 (14.3–14.9)
HCT (%)	7	35.7 (31.9–39.1)	36.9 (36.2–38.5)	36.7 (35.0–39.7)	36.9 (35.3–38.2)
	14	39.5^*^ (38.9–41.3)	38.9^*^ (37.1–41.9)	38.4 (30.9–42.0)	38.4 (36.2–40.4)
	28	40.1^*^ (38.8–41.4)	37.8 (34.2–41.3)	39.6^*^ (38.7–44.0)	38.9^**^ (37.9–40.0)
MCV (μm^3^)	7	59.0 (58.0–61.0)	59.0 (58.0–63.0)	60.0 (57.0–63.0)	60.0 (59.0–62.0)
	14	59.0 (58.0–60.0)	57.5 (57.0–61.0)	59.0 (56.0–61.0)	59.0 (59.0–61.0)
	28	56.0^*^^∧^ (55.0–58.0)	55.5^**^^∧^ (54.0–57.0)	57.0^**^ (54.0–59.0)	57.0^**^^∧∧^ (54.0–58.0)
MCH (pg)	7	22.8 (22.1–23.8)	22.4 (21.9–24.1)	23.1 (21.3–24.5)	23.4 (22.0–24.1)
	14	22.4 (21.8–23.5)	22.0 (21.3–23.6)	22.6 (20.7–23.5)	23.0 (22.6–23.6)
	28	21.1^**^^∧^ (20.5–21.9)	20.8^**^^∧∧^ (20.1–21.4)	21.3^**^^∧^ (19.9–21.9)	20.9^**^^∧∧^ (19.7–22.0)
MCHC (g/dl)	7	38.1 (37.4–38.7)	38.1 (37.4–38.3)	38.0 (37.6–39.4)	38.8 (36.7–39.7)
	14	38.2 (37.9–38,9)	38.1 (37.1–38.6)	38.2 (37.3–38.8)	38.9 (38.2–39.4)
	28	37.5 (36.4–38.3)	37.3 (35.3–38.3)	36.9^*^ (32.3–38.8)	37.3^*^^∧∧^ (36.0–37.8)
PLT (10^3^/mm^3^)	7	415.0 (390.0–546.0)	435.0 (366.0–591.0)	432.0 (361.0–637.0)	432.0 (380.0–658.0)
	14	486.0 (211.0–658.0)	418.0 (287.0–602.0)	426.0 (285.0–689.0)	581.0 (336.0–699.0)
	28	470.0 (406.0–537.0)	444.0 (395.0–490.0)	431.0 (323.0–571.0)	425.0 (311.0–545.0)

*PBS (vehicle) treated rats served as a control group*.

*WBC, white blood cell count; RBC, red blood cell count; HGB, hemoglobin; HCT, haematocrit; MCV, mean corpuscular volume; MCH, mean corpuscular hemoglobin; MCHC, mean corpuscular hemoglobin concentration; PLT, platelet count. Results are shown as median with lower and upper limits*.

* *p < 0.05*,

** p < 0.01 vs. 7 days;

∧ *p < 0.05*,

∧∧ *p < 0.01 vs. 14 days, Mann-Whitney test*.

### Influence of the Dex40-GTMAC3 on biochemical parameters

To examine the effect of Dex40-GTMAC3 on hepatic and renal functions we measured ALT and AST activities in the serum collected from rats that had been intravenously injected with heparin (300 U/kg) alone or followed by Dex40-GTMAC3 (7.5 mg/kg) or protamine (3 mg/kg) 6 h, 14, and 28 days before collection. PBS (vehicle) treated rats served as a control group. Additionally, ALP, AMYL, CPK, and CREA activities were measured. Blood biochemistry did not change in all time points (Table 2) and no statistically significant changes in measured parameters were found, compared with vehicle-treated rats. Similarly to blood count we found significant changes in ALT, ALP, AMYL, and CREA between time points within groups.

**Table 2:** **Biochemical analysis of serum samples from rats treated with UFH (300 U/kg) alone or followed by Dex40-GTMAC3 (7.5 mg/kg) or protamine (3 mg/kg)**.

**Parameters (unit)**	**Time**	**Vehicle (PBS)**	**UFH 300 U/kg**	**UFH 300 U/kg + Dex40-GTMAC3 7.5 mg/kg**	**UFH 300 U/kg + Protamine 3.0 mg/kg**
ALT (U/L)	6 h	44.0 (38.0–54.0)	46.0 (40.0–63.0)	48.0 (39.0–60.0)	49.0 (41.0–59.0)
	14 days	52.0 (43.0–68.0)	59.0^*^ (50.0–70.0)	57.0^*^ (51.0–63.0)	54.0 (48.0–58.0)
	28 days	38.0^∧∧^ (31.0–44.0)	37.0^**^^∧∧^ (28.0–49.0)	34.0^**^^∧∧∧^ (28.0–41.0)	38.0^**^^∧∧^ (31.0–41.0)
AST (U/L)	6 h	69.5 (62.0–104.0)	77.0 (54.0–88.0)	67.0 (48.0–93.0)	80.0 (50.0–113.0)
	14 days	67.5 (64.0–69.0)	73.0 (66.0–92.0)	67.0 (62.0–86.0)	65.0 (61.0–74.0)
	28 days	68.0 (58.0–78.0)	66.0 (57.0–81.0)	68.0 (49.0–85.0)	67.0 (54.0–82.0)
ALP (U/L)	6 h	328.0 (307.0–334.0)	377.0 (231.0–426.0)	288.0 (259.0–357.0)	302.5 (257.0–405.0)
	14 days	299.0 (367.0–445.0)	289.0 (204.0–340.0)	246.5 (211.0–399.0)	273.0 (217.0–388.0)
	28 days	149.0^*^^∧∧^ (132.0-178.0)	155.0^***^^∧∧∧^ (112.0–164.0)	128.0^**^ (110.0–175.0)	140.0^**^^∧∧^ (112.0–176.0)
AMYL (U/L)	6 h	831.0 (781.0–940.0)	877.0 (772.0–1080.0)	965.0 (845.0–976.0)	873.0 (843.0–937.0)
	14 days	862.0 (768.0–881.0)	801.0 (709.0–973.0)	841.5 (833.0–991.0)	874.5 (820.0–961.0)
	28 days	854.0 (816.0–892.0)	755.0 (740.0–999.0)	807.0^*^ (681.0–894.0)	818.0 (755.0–943.0)
CREA (mg/dL)	6 h	0.51 (0.43–0.51)	0.46 (0.43–0.53)	0.44 (0.42–0.46)	0.45 (0.44–0.45)
	14 days	0.45 (0.45–0.47)	0.47 (0.43–0.49)	0.47^**^ (0.46–0.49)	0.46 (0.45–0.47)
	28 days	0.52^∧^ (0.5–0.52)	0.49^∧^ (0.48–0.52)	0.49^**^ (0.48–0.52)	0.49^*^ (0.46–0.57)
CPK (U/L)	6 h	416.5 (321.0–512.0)	419.0 (179.0–631.0)	343.0 (196.0–772.0)	467.5 (183.0–667.0)
	14 days	344.0 (252.0–687.0)	399.5 (277.0–550.0)	284.0 (206.0–531.0)	312.0 (226.0–425.0)
	28 days	387.5 (249.0–588.0)	270.0 (127.0–817.0)	396.5 (168.0–515.0)	341.0 (234.0–676.0)

*PBS (vehicle) treated rats served as a control group*.

*AST, aspartate transaminase; ALT, alanine transaminase; CREA, creatinine; AMYL, amylase; ALP, alkaline phosphatase; CPK, creatine kinase. Results are shown as a median with lower and upper limits*.

* *p < 0.05*,

** *p < 0.01*,

*** p < 0.001 vs. 6 h;

∧ *p < 0.05*,

∧∧ *p < 0.01*,

∧∧∧ *p < 0.001 vs. 14 days, Mann-Whitney test*.

### Organs morphology

**Figure 8:**
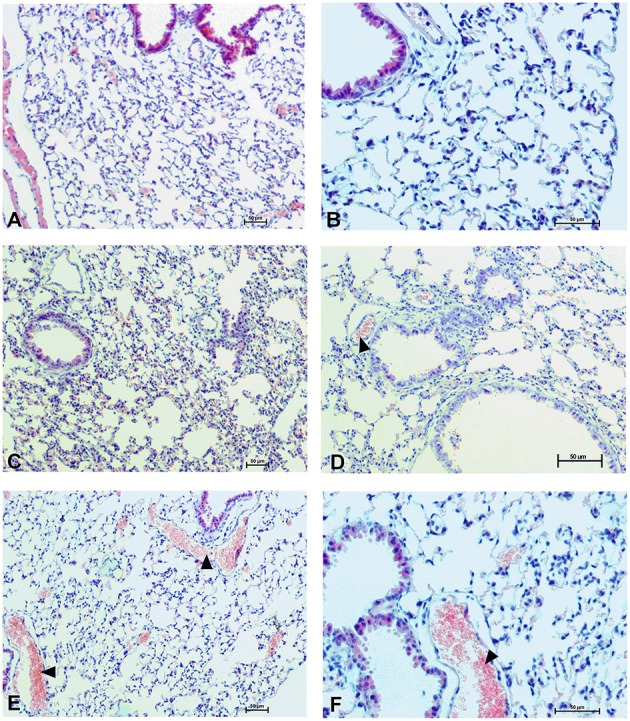
**Pulmonary changes in the male mice 1 h after drug administration**. Photomicrographs of histological sections of lungs from mice after 1 h of treatment with UFH 300 U/kg **(A,B)**, UFH + Dex40-GTMAC3 7.5 mg/kg **(C,D)**, and UFH + protamine 3 mg/kg **(E,F)**. Images (**A,C,E)** were taken using the 100 × magnification and images **(B,D,F)** were taken using 200 × magnification. Arrowheads indicate congestion.

**Figure 9:**
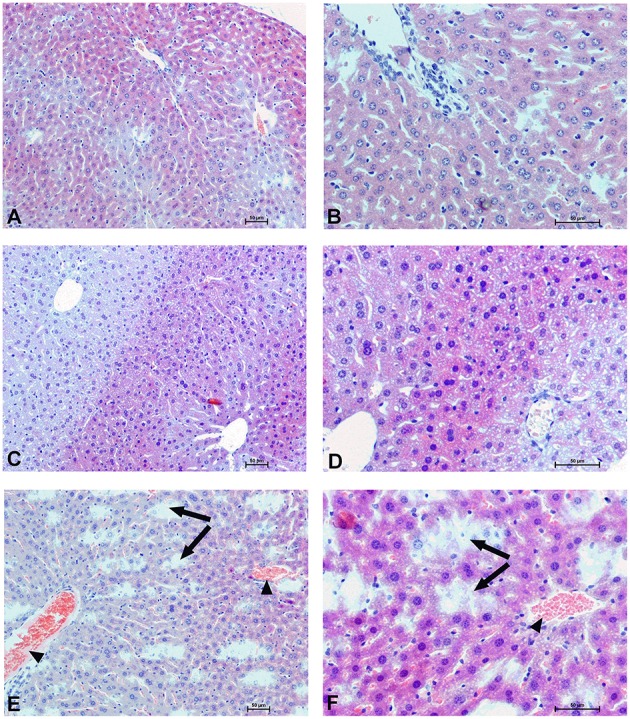
**Hepatocellular changes in the male mice 1 h after drug administration**. Photomicrographs of histological sections of the mouse liver isolated 1 h after the administration of UFH 300 U/kg **(A,B)**, UFH + Dex40-GTMAC3 7.5 mg/kg **(C,D)**, and UFH + protamine 3 mg/kg **(E,F)**. Images **(A,C,E)** were taken using the 100 × magnification and images **(B,D,F)** were taken using 200 × magnification. Arrowheads indicate congestion; arrows indicate necrotic foci.

**Figure 10:**
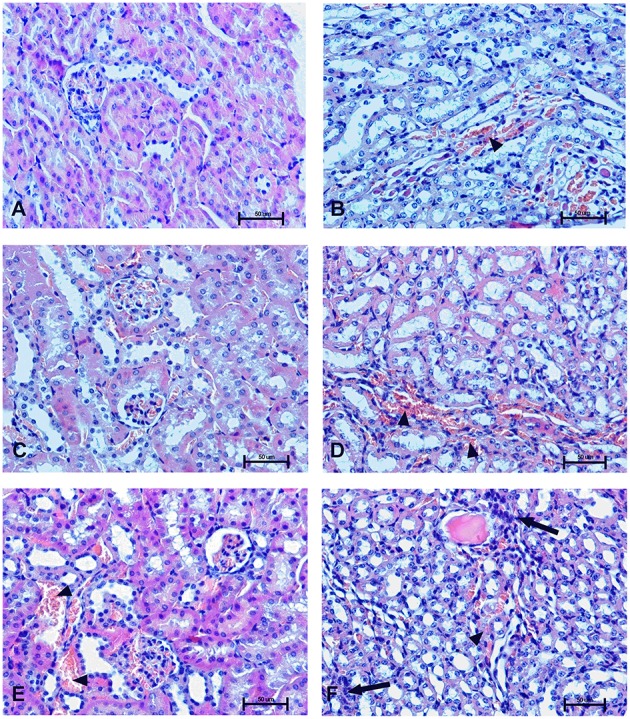
**Changes in cortical (A,C,E) and indigenous (B,D,F) kidney in the male mice 1 h after drug administration**. Photomicrographs of histological sections of lungs from mice isolated 1 h after the treatment with UFH 300 U/kg **(A,B)**, UFH + Dex40-GTMAC3 7.5 mg/kg **(C,D)**, and UFH + protamine 3 mg/kg **(E,F)**. Images were taken using 200 × magnification. Arrowheads indicate congestion; arrows indicate infiltration of inflammatory cells.

**Figure 11:**
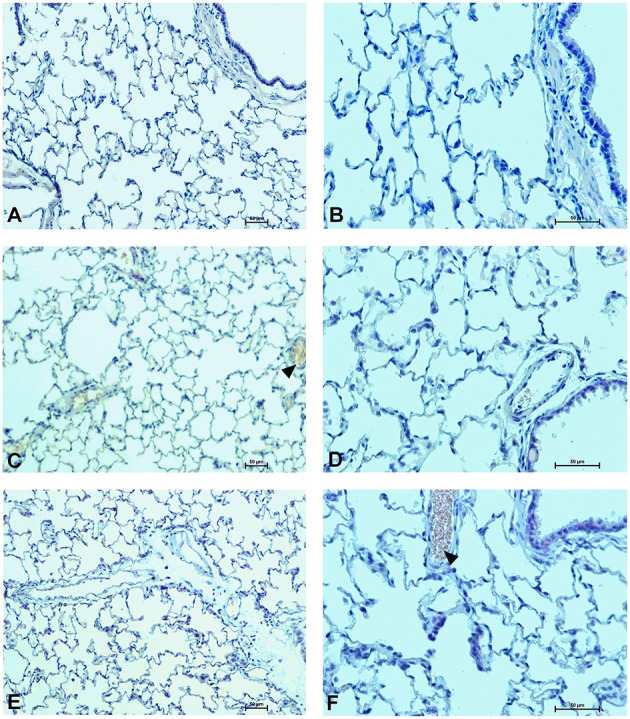
**Pulmonary changes in the male rats observed 28 days after the treatment**. Photomicrographs of histological sections of lungs isolated from rats 28 days after the administration of UFH 300 U/kg **(A,B)**, UFH + Dex40-GTMAC3 7.5 mg/kg **(C,D)**, and UFH + protamine 3 mg/kg **(E,F)**. Images **(A,C,E)** were taken using the 100 × magnification and images **(B,D,F)** were taken using 200 × magnification. Arrowheads indicate congestion.

**Figure 12:**
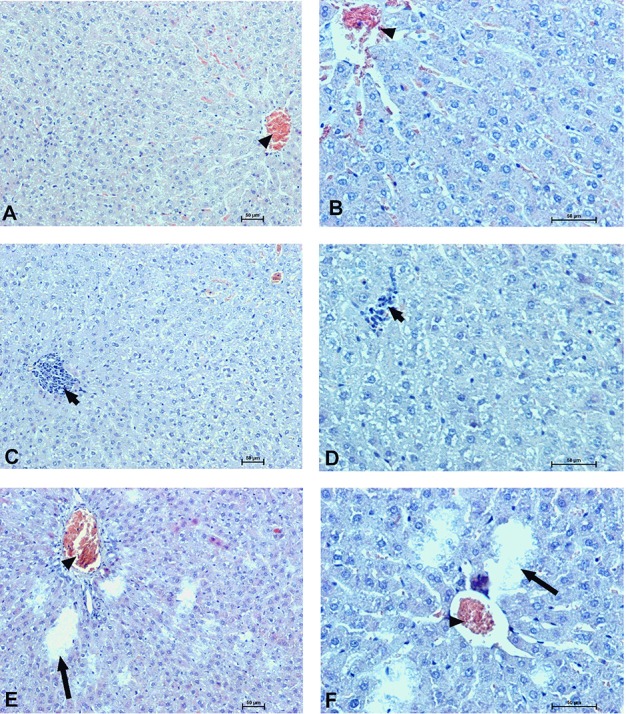
**Hepatocellular changes in the male rats observed 28 days after the treatment**. Photomicrographs of histological sections of the liver isolated from rats 28 days after administration of UFH 300 U/kg **(A,B)**, UFH + Dex40-GTMAC3 7.5 mg/kg **(C,D)**, and UFH + protamine 3 mg/kg **(E,F)**. Images **(A,C,E)** were taken using the 100 × magnification and images **(B,D,F)** were taken using 200 × magnification. Arrowheads indicate congestion; short arrows indicate infiltration of inflammatory cells; long arrows indicate necrotic foci.

**Figure 13:**
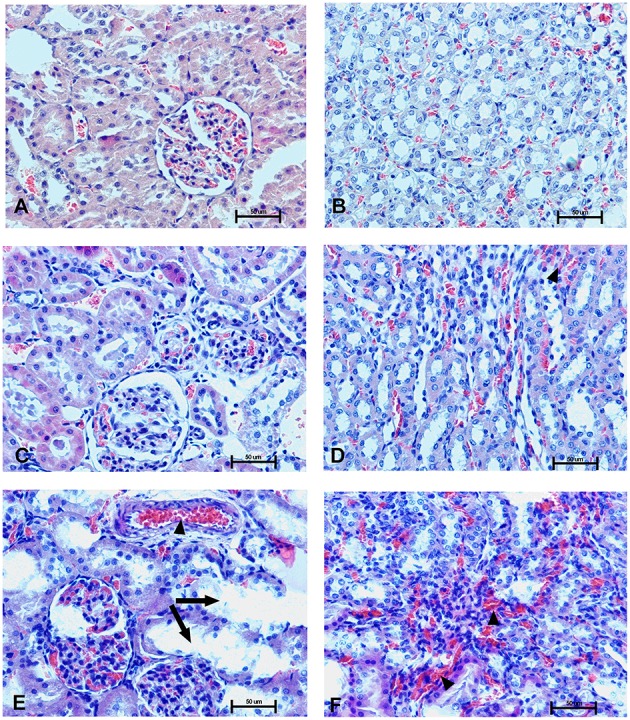
**Changes in the cortical (A,C,E) and indigenous (B,D,F) kidney in rats 28 days after the drug administration**. Photomicrographs of histological sections of kidneys isolated from rats 28 days after the administration of UFH 300 U/kg **(A,B)**, UFH + Dex40-GTMAC3 7.5 mg/kg **(C,D)**, and UFH + protamine 3 mg/kg **(E,F)**. Images were taken using 200 × magnification. Arrowheads indicate congestion; arrows indicate necrotic foci.

To determine the effect of Dex40-GTMAC3 and protamine on the rodent tissues, we examined liver, lungs and kidneys histology 1 h (mice) and 28 days (rats) after the termination of the experimental procedure. Routine histopathological examination showed normal liver, kidneys and lungs morphology of the PBS-injected animals in both groups, sacrificed 1 h and 28 days after vehicle administration. Specimens of the examined organs in both groups of animals did not show any pathological features. Though the image of the lung, kidney and liver lobules construction of UFH-treated animals was preserved, yet 1 h after UFH injection, the analysis of lung showed slight congestion of intra-acinar vessels and eosinophil-bronchial epithelial cell interaction (Figures 8–13).

In microscopic examination histological changes were seen in the lungs, liver, and kidneys of rats treated with Dex40-GTMAC3 in acute experiment. Histopathologically, lesion characterized by engorgement of blood vessels and an increase of eosinophilic cytoplasm some cells of the lungs, liver and kidneys. Liver histology of rats in acute Dex40-GTMAC3 intoxication showed little vacuolar degeneration of hepatocytes and local leukocytic infiltration around central vein. Morphological evaluation of the lungs of rats receiving Dex40-GTMAC3 showed moderate vascular and interstitial lung congestion and some epithelial cells lining the airways showed strongly eosinophilic cytoplasm. There was slight vacuolization of proximal tubular cells. It appears that these alterations are reversible. Twenty eight days after Dex40-GTMAC3 injection we still observed slight vacuolation and ballooning enlargement of hepatocytes with preserved central location of the cell nucleus (Table 3). Also, lymphocytes were locally found within hepatic parenchyma without associated cellular necrosis (Figures 8–13).

**Table 3:** **Summary of notable histological abnormalities in the liver, lungs, and kidneys of rats recorded 28 days after drug administration**.

	**Vehicle (PBS)**	**UFH 300 U/kg**	**UFH 300 U/kg + Dex40-GTMAC3 7.5 mg/kg**	**UFH 300 U/kg + Protamine 3 mg/kg**
Lungs (total examined)	6	7	7	7
Lung congestion (n)	0	4	6^*^	7^*^
Liver (total examined)	6	7	7	7
Hepatocelullar vacuolation (n)	0	0^∧^	7^*^	0^∧^
Hepatocellular fatty vacuoles (n)	0	0	0	7^*^^, ∧^
Hepatocellular necrotic foci (n)	0	0	0	7^*^^, ∧^
Kidneys (total examined)	6	7	7	7
Vacuolation of tubular cells (n)	0	0^∧^	7^*^	7^*^

*PBS (vehicle) treated rats served as a control group*.

* *p < 0.05 vs. PBS*;

∧ *p < 0.05 vs. UFH 300 U/kg + Dex40-GTMAC3 7.5 mg/kg, Fisher exact test with post-hoc Holm p-value adjustment*.

In contrast to the UFH- and PBS-treated groups, the histological outline of the lungs in the protamine-treated group revealed strong alveolar hemorrhage. The enlargement of the alveolar sacs was also found. We noticed faded lobular structure of the liver with necrotic foci. In the cytoplasm of hepatocytes we found numerous fatty vacuoles corresponding to the fatty degeneration. There were also erythrocytes in blood vessels and the liver sinusoids. Necrotic lesions were also observed 28 days after administration of protamine. In kidneys we observed vacuolation and sometimes complete exfoliation of epithelial cells lining the tubular part of the nephron. Around the renal corpuscle and along the blood vessel infiltration of inflammatory cells could be observed. In some animals in the study group histopathological changes were very severe, including a noticeable damage of the cell nuclei (Figures 8–13, Table 3).

## Discussion

During the past six decades dextrans have been extensively used as plasma volume expanders because of their high biocompatibility and biological inertness resulting from their uncommon poly-(α-D-1,6-glucose) linkage. We previously reported that 40 kDa dextrans with the degree of substitution with cationic groups of 0.5 (Dex40-GTMAC2) and 0.65 (Dex40-GTMAC3) per glucose unit effectively reversed anticoagulant and antithrombotic action of heparin in rodents without inducing immunogenic response (Kalaska et al., 2012, 2015). However, Dex40-GTMAC2 given alone in a therapeutic dose significantly decreased blood pressure, whereas Dex40-GTMAC3 did not, but further escalating of dose revealed similar hypotensive properties (Kalaska et al., 2015). Thus, we aimed to estimate the body distribution and potential toxicity of Dex40-GTMAC3 to further explore its pharmacological effects.

In the present study we demonstrated that Dex40-GTMAC3 displays a favorable to protamine safety profile in rodents. We found fast renal clearance behavior and low tissue accumulation of Dex40-GTMAC3. New heparin reversing agent was very well tolerated by rats up to 28 days after single intravenous injection: body weight, blood count and blood biochemistry remained unchanged. Acute and long-lasting histological alterations in lungs, kidneys and liver of heparinized rats and mice were less pronounced in Dex40-GTMAC3 treated group in comparison to protamine. Additionally, we showed that single injection of protamine might lead to permanent damage of liver and kidneys.

We found that fluorescein-labeled Dex40-GTMAC3 is rapidly eliminated from the rat plasma with the circulation half-time about 12 min. If we calculated the half-time based on the body mass-to-surface area ratio (37 for humans and 6 for rats; Regan-Shaw et al., 2008), it could be 6 times longer in humans. Half-time of unmodified dextran used as volume expander with a mean molecular weight of 40 kDa varies between 2 and 9 h in humans (Klotz and Kroemer, 1987; Tobias, 2005; Smith et al., 2011). High deviation is probably related to the fact, that dextran 40 kDa is a heterogeneous mixture of different chains weighting from 10 to 80 kDa (Mehvar et al., 1994). Interestingly, protamine half-time is very short and similar in humans and rats. In animals it is around 24 min in the absence and 18 min in the presence of UFH (Delucia et al., 1993), whereas in humans it is approximately 7.4 min (Butterworth et al., 2002).

We used Carestream *In Vivo* MS FX PRO® system, to monitor location of labeled-Dex40-GTMAC3 fluorescence signal in the living mouse. Similarly to unsubstituted dextrans (Mehvar et al., 1994), we found the highest concentration of Dex40-GTMAC3 in the liver and kidneys of rats and mice. In humans about 70% of an intravenous dose of unmodified 40 kDa dextran is excreted by kidneys within 24 h. The remaining 30% is retained or metabolized (Howard et al., 1955) by different exo- and endodextranases (α-1-glucosidases) present at higher concentration in the liver (Lake et al., 1985). In comparison, protamine given intravenously distributes rapidly to kidneys, lungs, and heart (Delucia et al., 1993).

We used commercially available fluorophores covalently attached to the tested polymer to determine Dex40-GTMAC3 concentration in the biological samples. Although, it can be easily detected in fluorometer, it does not allow distinguishing between the parent macromolecule and its possible degradation products. Negatively charged fluorescein theoretically could also interfere with hepatic kinetics of Dex40-GTMAC3/UFH complexes. On the other hand the substitution of Dex40-GTMAC3 with fluorescein was very low. We also used second fluorophore—rhodamine B to confirm organ distribution of Dex40-GTMAC3. Based on this method, we think that the body distribution and elimination of cationically-modified dextrans is similar to that of unmodified dextrans (Larsen, 1989; Burns et al., 1995; Mehvar, 2000; Dhaneshwar et al., 2006). The fast renal clearance behavior and generally low tissue accumulation of Dex40-GTMAC3 is likely to contribute to its superior to protamine biocompatibility.

Because of the cationic nature, protamine may disrupt erythrocyte membrane and induce hemolysis by an interaction with the anionic surface of a cell (Carroll et al., 1959), which may reside in the lipoprotein component (Becker, 1961). The molecular weight and the balance between hydrophobic and hydrophilic groups of cationic polymers prompted us to test their potential blood toxicity, although we did not previously observe significant changes under microscope (Kaminski et al., 2010). Thus, before performing *in vivo* studies we assayed Dex40-GTMAC3 for potential hemolytic effects to compare them with those of protamine. We found small (not more than 3%) difference in erythrocyte osmotic resistance after incubation with both agents, in slight favor of Dex40-GTMAC3, because protamine induced statistically significant change in the lowest concentration. In fact, Dex40-GTMAC3 effect was opposite to that of protamine. Others found protective rather than destructive effect of unmodified dextrans on the erythrocyte membrane (Cudd et al., 1989).

Dex40-GTMAC3 will be typically administered to patient as single injection. Therefore, we evaluated its safety in different time points up to 28 days after single intravenous administration of Dex40-GTMAC3 followed by UFH. Animals' behavior and body weight were the same as control or UFH treated group and we did not observe any changes in the blood count and in the clinical biochemistry during the whole experiment.

Based on our body distribution data we examined the histopathological changes in kidney, liver and lungs of heparinized mice 1 h and 2 days after single administration of 1 ml/kg PBS solution of Dex40-GTMAC3 (0.75%) or protamine (0.3%). We did not examine spleen in detail, because no significant differences in spleen tissue were previously detected in repeated-dose 1 month long experiment in mice (Kalaska et al., 2015). In Dex40-GTMAC3-treated group we observed slight vacuolization of proximal tubular cells. Intravenous administration of dextrans was shown to cause reversible renal abnormalities, characterized by vacuolization of proximal tubular cells in human kidneys (so-called osmotic nephrosis; Vos et al., 2002). Kitazawa et al. (2014) reported changes in liver, lungs, spleen and kidneys, when treated rats with 10% saline solution of 40 or 200–300 kDa dextrans in dose of 5 ml/kg/day for 28 days. Daily dose used in that study was much higher than a dose of Dex40-GTMAC3 used in our study to neutralize high dose of UFH (prolonging activated partial thromboplastin time more than 15 times). Osmotic nephrosis-like lesions were reported for other biopolymers such as hydroxyethyl starches (Legendre et al., 1993) and gelatins (Kief, 1969). They were also observed with agents that do not induce acute renal failure such as mannitol and glucose (Kief, 1969).

Nashida et al. (1991) showed that the hepatic disposition of neutral and anionic dextrans are similar. Therefore, we also expected changes in the liver in the case of cationic dextrans, especially because positively charged macromolecules are rapidly taken up by the liver (Nakane et al., 1988; Nishida et al., 1990) and enter the hepatic cells by fluid-phase endocytosis (Lake et al., 1985; Stock et al., 1989). We noticed cytoplasmic vacuolation of hepatocytes 1 h after drug administration. We found that the vacuolated hepatocytes in animals are not damaged, but they probably represent cells regenerating in response to hepatic injury. Nayak et al. (1996) found that the hepatocytes with non-lipid cytoplasmic vacuolization seen in acute and subacute liver injury are cells that have adapted to the toleration of further injury. Their vacuolation seems to be a relatively short-lived, acute-phase adaptive response to milder forms of injury. Some hepatocytes showed enlargement of both hepatocyte cytoplasm and nuclei, not typically seen in control mice. Spontaneous occurrence of karyocytomegaly is more common in aging mice than in rats (Lu et al., 1993; Styles, 1993) and it is not typically associated with increased liver weight. We did not observe such changes in rats.

Long-term response to single injection of Dex40-GTMAC3 or protamine to rats was analyzed in tissue sections from kidney, liver, and lungs collected at 28th day after administration. We did not observe detectable tissue damage, such as cell necrosis or leukocyte infiltration in the organs from both control and Dex40-GTMAC3 treated rats. The vacuolization of hepatocytes and proximal tubular cells seemed to be less pronounced after 28 days in comparison to the acute response. Others also found reversible vacuolization of hepatocytes induced by unmodified dextrans in rats (Mehvar et al., 1994).

Interestingly, we found signs of organ damage 28 days after single injection of protamine, presented as strong congestion, lipid vacuoles and necrosis of hepatocytes with nuclei degradation. Several authors have reported that protamine may inhibit hepatic lipase activity (Harwood et al., 1974; Berger and Abraham, 1977) or cause nephrosis resulting in hypercholesterolemia (Saito et al., 1987). We also found vacuolation and sometimes complete exfoliation of epithelial cells lining the tubular part of the nephron. Protamine is known to have an adverse effect on the systemic circulation. Injection of protamine resulted in capillary thrombosis and severe damage to both glomerular and tubular epithelium (Messina et al., 1989; Kurihara et al., 1992). Although the mechanism of protamine nephrotoxicity is unclear, even small dose may be toxic to the cellular components by neutralizing anionic sites in the glomerulus and cause reversible epithelial damage and change of renal function (Andrew, 1978; Vehanskarl et al., 1982; Adler et al., 1983; Messina et al., 1989).

We also found diffusive intra-alveolar foam cell aggregation in the lungs of the animals 1 h after injection of protamine. Similarly, others reported direct toxic effect on isolated rat lungs (Fairman et al., 1987) and in living animals, presented as distinct hemorrhage, pulmonary edema, and inflammatory cell infiltration (Koslow et al., 1987; Cook et al., 1992). The mechanism of acute pulmonary toxicity probably involves activation by UFH-protamine complexes the classical complement pathway leading to the formation of C5a, which causes leukoaggregation and leukoactivation, release of oxygen free radicals and lipid peroxidation (Morel et al., 1988). Taking together our and others results the release of thromboxane A2 by intravascular macrophages (Chang and Voelkel, 1989) leading to vasoconstriction and activation of blood platelets in lungs (Jenkins et al., 1971; Eika, 1972) may be the main cause of protamine's severe adverse effects in patients, such as pulmonary hypotension and bradycardia. Our results put one more red flag on the use of this antidote and signal the need for long-term monitoring of patients, especially receiving repeatable injections of protamine.

In contrast to protamine-treated animals we observed only slight congestion of the lungs after administration of Dex40-GTMAC3. We found one report of noncardiogenic pulmonary edema in previously healthy patient receiving dextran 40 kDa (Kaplan and Sabin, 1975), but such pulmonary capillary leakage and subsequent pulmonary edema were not included among the adverse reactions of dextran 40 kDa (Data and Sies, 1974).

Our previous study (Kalaska et al., 2015) showed that Dex40-GTMAC3 effectively reverses heparin-induced anticoagulation without producing any of the acute adverse effects exhibited by protamine. Moreover, it did not induce immune system response in contrast to protamine (Bakchoul et al., 2013), heparinase I (Stafford-Smith et al., 2005), lactoferrin (Wu et al., 1995) or low molecular weight protamine (Chang et al., 2001). The safety of other alternative UFH antidotes such as haxadimetrine bromide (Ransdell et al., 1965), methylene blue (Kikura et al., 1996; Ginimuge and Jyothi, 2010), vancomycin (Kikura et al., 1996), lactoferin (Wu et al., 1995), or chemically-modified inactive antithrombin (Fazavana et al., 2013) were not sufficiently examined in animals and they all failed in the later phases of drug discovery. Our present study provides toxicokinetic profile of Dex40-GTMAC3—a potential UFH reversal agent. The research and development costs for Dex40-GTMAC3 should be also significantly lower than those of protein-based antidotes such as protamine or andexanet alfa. Our histopathology results in the protamine-treated animals indicate the need for closer observation of its long-term effects, especially in patients with liver or renal insufficiency.

Taking together all of our studies on the cationically-modified dextrans, we are introducing here Dex40-GTMAC3—a new highly effective and safer than protamine candidate for the reversal of unfractionated heparin. We are aware that the safety in rodents and humans may differ and our results need to be verified on larger mammals before entering clinical trials. We hope that superior to protamine pharmacological profile of Dex40-GTMAC3 will address the unmet need for a new efficient and safer antidote of UFH.

## Author contributions

Conceived and designed the experiments: ES, BK, KK, KS, MN, DP, JW, IK, AM. Performed the experiments: ES, BK, AL, AB, KK, KS, AM. Analyzed the data: ES, BK, KK, AB, KS, MN, IK, JW, DP, AM. Contributed reagents/materials/analysis tools: KK, KS, MN. All authors took part in drafting the work, revising it critically and approved all parts of the work.

## Funding

The study was supported by National Science Centre Grant No. DEC-2011/03/B/NZ7/00755. BK was supported by funds from Leading National Research Center in Bialystok (110/KNOW/2015). KK was supported by National Science Centre Grant No. UMO-2013/09/D/ST5/03864. ES was supported by funds from “Studies, research, commercialization - a support programme of UMB doctoral students” Sub-measure 8.2.1 Human Capital Operational Programme, co-financed from the European Union under the European Social Fund.

### Conflict of interest statement

The authors declare that the research was conducted in the absence of any commercial or financial relationships that could be construed as a potential conflict of interest.
